# Promoting Sustained Breastfeeding of Infants at Risk for Asthma: Explaining the “Active Ingredients” of an Effective Program Using Intervention Mapping

**DOI:** 10.3389/fpubh.2018.00087

**Published:** 2018-03-20

**Authors:** Ilse Mesters, Barbara Gijsbers, L. Kay Bartholomew

**Affiliations:** ^1^Department of Epidemiology, Care and Public Health Research Institute (CAPHRI), Maastricht University, Maastricht, Netherlands; ^2^Department of General Practice, Care and Public Health Research Institute (CAPHRI), Maastricht University, Maastricht, Netherlands; ^3^Health Promotion and Behavioral Sciences, University of Texas School of Public Health, Houston, TX, United States

**Keywords:** breastfeeding, educational program, asthma, Intervention Mapping, program theory

## Abstract

Infants whose parents and/or siblings have a history of asthma or allergy may profit from receiving exclusive breastfeeding during the first 6 months of life. This is expected to diminish the chance of developing childhood asthma and/or atopic disease. Ongoing breastfeeding for 6 months seems challenging for many women. An educational program was developed using Intervention Mapping as a logic model to guide development and was found successful in improving breastfeeding rates at 6 months postpartum, improving knowledge and beliefs about breastfeeding for 6 months, after exposure to the program compared to controls. Intervention elements included an evidence- and theory-based booklet addressed during pre- and postnatal home visits by trained assistants. This paper elucidates the inner workings of the program by systematically describing and illustrating the steps for intervention development.

## Introduction

Complex behavior change interventions need evidence regarding the effectiveness of individual components to understand how these interventions work. The objective of this paper is to identify, guided by the Intervention Mapping (IM) protocol, the effective elements of an existing educational program, which were shown to be effective in increasing exclusive breastfeeding.

Exclusive breastfeeding during the first 6 months after birth is expected to diminish the chance of developing childhood asthma and/or atopic disease (1, 2). This effect may be particularly apparent in familial predisposed children (3, 4). Unfortunately, exclusive breastfeeding (entailing avoidance of solid foods^1^) appears to be a difficult behavior for women to perform and continue for 6 months. Only 18% of Dutch women succeeded in doing so in 2010 (7). In recent years, many programs have been developed to increase breastfeeding initiation and duration rates, employing diverse methods and theories, with variable results (8). Our multifaceted, theory-informed breastfeeding program, which combined two pre- and one postnatal home visits and a booklet, appeared to be effective in promoting exclusive breastfeeding for at least 6 months in asthmatic families in 2005 (7). The intervention group, compared to the control group, entailed a significantly higher proportion of women that breastfed exclusively at 6 months, 48 versus 27%, respectively, providing evidence that written and oral advice about exclusive breastfeeding was effective in improving the exclusive breastfeeding rates at 6 months in asthmatic families. Moreover, significant improvements in knowledge and more positive beliefs regarding ongoing breastfeeding for 6 months were revealed in the intervention group compared with the controls, particularly directly after contact with the program. As anticipated, perceived self-efficacy and women’s positive emotions toward breastfeeding increased and support for breastfeeding diminished in both groups over time. The intervention group reported perceiving more pressure to bottle feed and to be aware of more breastfeeding models than the control group (9).

**Figure 1:**
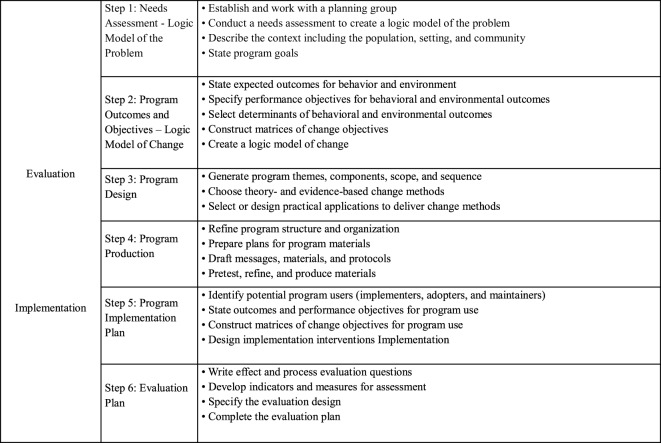
Intervention mapping steps and tasks [adapted from Bartholomew et al. (12), p. 13].

Like educational or health promotion programs for other problems, it is difficult to know what parts of these programs have contributed to effectiveness or ineffectiveness. Failure to report adequately about the program theory, theory- and evidence-informed behavior change techniques, and practical delivery strategies hampers the growth of the science of health promotion. A number of researchers have called for better reporting (10, 11). Program development and description guided by IM (12) may help to explain what is “inside the black box” (13). IM has not only been used to develop effective health promotion programs but also to describe the intervention content (14, 15), see Figure 1. IM distinguishes six planning steps, with each step comprising several tasks.

In the first step, the planner puts together a planning group to assess the health problem, the behavioral and environmental factors influencing the health problem, and determinants of behavioral and environmental causes, which are then depicted in a logic model of the health problem. Step 2 specifies program outcomes and objectives in a logic model of change. In step 3, a coherent, deliverable intervention is designed. Theory-based intervention methods and practical applications to change (determinants of) behavior are selected, and program themes, components, scope, and sequence are generated. Step 4 comprises the actual production of the program. In step 5, a program implementation plan is generated. In step 6, a plan is generated for effect and process evaluations. Activities for steps 5 and 6 start as early as possible in the planning process. Although IM is presented as a sequence of actions, the authors see the planning process as iterative rather than chronological.

In this article, we use IM to describe the successive steps of the program development, especially the theoretical change methods and practical delivery applications used in our program to promote breastfeeding.

## Methods

### Setting and Priority Population

The intervention described in this report and its evaluation was a sequel to a Dutch prospective birth cohort study called PREVASC (16). In this cohort, families had been advised on a variety of asthma control behaviors including breastfeeding to prevent asthma (and allergies) in high-risk infants. The primary care program further included hypoallergenic feeding as an alternative to breastfeeding, postponement of solid food, the introduction of house dust mite impermeable bed coverings, and smoking cessation. Mothers were included when 3–7 months pregnant. Adherence to the breastfeeding advice was low, a finding that called for refinement of the breastfeeding intervention.

### Intervention Development

Intervention mapping guided the identification of determinants of breastfeeding (and postponement of solid foods), the formulation of intervention objectives, the choice of methods and practical applications for inducing a change in determinants and feeding behaviors, and creation of ideas for program implementation and evaluation. In this article, the first four IM steps will be illustrated in more detail.

1. *Needs assessment: the logic model of the problem*. Improvement of breastfeeding adherence was the interest of this project. Our needs assessment comprised two studies: a review of the literature and a qualitative study (17). Both studies aimed at increasing our understanding of the determinants both of early discontinuation of breastfeeding and of maintaining breastfeeding for 6 months. For the literature review, we searched Psychlit and Medline for publications on breastfeeding interventions and determinants of breastfeeding. The qualitative study entailed seven focus group interviews to explore the breastfeeding-related behaviors within 43 families in which either one of the parents and/or one of the children had physician-diagnosed asthma. Both successful and unsuccessful families in breastfeeding for 6 months were randomly selected from the Prevask study described above. There were six mixed groups (36 participants, 36 mothers, and 14 fathers) and one group consisted of solely fathers (7 participants), since they may think differently about breastfeeding and postponement of solid food, thoughts which might be overlooked in the presence of women.
2. *Program outcomes and objectives: the logic model of change*. We developed the program plan by specifying who and what will change as a result of the intervention. We combined performance objectives for each target group (women and partners) with chosen determinants to produce change objectives, the most proximal focus of an intervention.
3. *Program design*. Based on the change objectives, we sought theory-based methods and practical applications to change the factors that influence the target behaviors. An intervention method is a technique or procedure that is expected to change one or more factors that influence a target behavior of individuals, groups, or social structures while a practical application concerns the way methods are delivered to fit the context of the target population. We created a sketch of the program elements, their purpose and arrangement, the final program materials, and the program protocols.
4. *Program production*. All program components were pretested. We did this by presenting the booklet to eight women, by means of the plus–minus method. The plus–minus method involved asking participants to read the booklet from beginning to end and to indicate their positive and negative experiences in the margin with pluses and minuses, respectively. Pluses and minuses may be assigned to all sorts of text elements (from chapters to words) and for various reasons (e.g., comprehensibility, acceptability, interest, the relevance of the information). After that, we interviewed the participants to elicit the reasons for every plus and minus. We followed the interviews with a short semistructured questionnaire focusing on the macrostructure and a general evaluation of the text in the brochure (18).
5. *Program implementation plan*. This step includes program adoption and implementation (including consideration of program maintenance). We did not perform this step here because the program was implemented as a part of a research study by research personnel (efficacy study). Implementation after the RCT will focus on professionals and organizations specialized in pregnancy and/or breastfeeding, e.g., midwives and gynecologists, who have personal contact with pregnant women, which is important since preparation to breastfeed during pregnancy is considered prerequisite. These potential intermediaries will be approached to discuss their role in this breastfeeding program. In this way, providing the intervention materials to the target population and subsequent dialog occurs during health provider contacts and best resembles the RCT context.
6. *Evaluation plan*. The evaluation consisted of assessing the impact of the program provided on behavior adherence, exclusive breastfeeding for 6 months, using diaries as a source for the behavioral outcome in survival analysis. Furthermore, changes in related determinants of exclusive breastfeeding were assessed by a determinant-questionnaire filled-out by participants before and after the preventive program was provided and was compared with determinants in the control group. The analysis was performed to examine distal program objectives (e.g., knowledge, attitude, social pressure/support, self-efficacy, emotions). Process evaluation criteria were formulated (e.g., barriers to recruitment, participant maintenance in program and data collection, exposure to materials, and extent of reading them).

## Resulting Program

### Step 1: Logic Model of the Problem

The needs assessment focused on barriers and facilitators to providing exclusively breast milk to newborns the first 6 months after birth. Thulier and Mercer (19) reviewed demographic biological and social variables associated with breastfeeding duration. Influential demographic factors that seemed beneficial for breastfeeding were non-black race, relatively older age, being married, higher educational level, higher social class, less exposure to free samples, and (active) distribution of formula. Biological variables supporting breastfeeding consisted of sufficient milk supply (enabling late introduction of solid food), no infant health problems (e.g., no preterm birth, no hospitalized infant), normal (pre-pregnant) maternal BMI, absence of physical challenges of breastfeeding (e.g., sore nipples, mastitis), maternal nonsmoking, multiparity/prior breastfeeding experience, and vaginal delivery. Beneficial social variables entailed the return to paid work after 6 months, positive family support (e.g., from fathers), and skilled professional support. Maternal knowledge, positive intention, interest, and enough confidence in breastfeeding were relevant psychological variables positively associated with breastfeeding.

The focus group interviews suggested that threats to the continuation of breastfeeding vary by three time periods—the prenatal preparatory phase, the postnatal initiation phase, and the postnatal continuation phase. In the prenatal phase, a woman and her partner may or may not have learned how to correctly breastfeed their child. Some parents became breastfeeding “experts” in solving breastfeeding problems that turned up during the initial 6 months after birth while others did not gain the knowledge and skills to deal with problems that arose after the birth. Interviews revealed that looking for solutions only happened when problems had occurred, which often resulted in breastfeeding failure since child feeding could not be postponed. Parents stated that they had lacked the self-confidence to stand up to “discouraging advice” from family and friends. Parents had to practically obtain a “fighting spirit” to maintain breastfeeding for 6 months.

The next critical period started after delivery. Certain problems, such as difficulty helping the baby to latch-on, sore nipples, and worries over whether the baby was feeding adequately, undermined efforts to breastfeed. Parents, who learned about breastfeeding prior to the birth and had been mentally exposed to thinkable risk scenarios, were better prepared to cope with such situations. In addition, parents talked about a strong perceived social pressure/criticism against breastfeeding and the postponement of solid food. This was considered a risk situation one really needed to be prepared for since this hindrance appeared rather unexpected. For the women, it was very important that their partners supported them.

The final precarious period was the return to work. Many women reported they did not prepare very well for the tasks during this period. For example, the mothers did not plan ahead with arranging for a breast pump and a room at work to express milk. Women who were not aware of these tasks and who did not know of the law regarding expressing milk during work time seemed to have been more vulnerable to quitting prematurely.

### Step 2: Program Outcomes and Objectives; Logic Model of Change

Based on the needs assessment, we formulated four main program objectives regarding breastfeeding and postponement of solids until 6 months: Women will 1. Give exclusive breastfeeding for 6 months; 2. Postpone solid food for 6 months; 3. Recognize social pressure and cope with it; and 4. Recognize risk situations and cope with them. From these objectives, we developed performance specifications. These overall objectives were split into intermediate objectives such as acquiring information by reading the booklet as a preparatory action before delivery. We then combined these performance objectives with the determinants of breastfeeding that were under control of the mother and partner, and subject to change. Other, environmental factors, such as social norms or social support may directly influence the desired health behavior as well, but they are in this setting very difficult to address. We addressed only one relevant external agent, the newborn’s father; a key person in breastfeeding support.

As an alternative to making other external agents direct targets of the program, we sought to address these environmental factors through change objectives for the women. For example, the social influence by grandmothers was dealt with through a change objective for the women dealing with perceived social influence and a change objective for the partner to support the mother when she is confronted with opinions against breastfeeding.

The performance objectives are mentioned in Table 1 for the mother and Table 2 for the partner combined with corresponding determinants. The cells in the matrix were populated by asking what needed to change in each performance objective for the mother or partner to accomplish the performance objective. These matrixes provided the map for developing intervention strategies. After we defined change objectives, the next step was to identify theoretical methods and practical applications that could be expected, according to theoretical and empirical evidence, to influence the change objectives from the matrices in Step 2 (20).

**Table 1:** Partial matrix of change objectives for women that exclusively breastfeed for 6 months (6 m-EBF).

Performance objectives before delivery	Knowledge	Skills and self-efficacy	Attitude	Perceived norm
The mother will				
Acquire information to prepare for 6 m-EBF from the booklet	Describe common misconceptions about 6 m-EBF Describe the relevance of 6 m-EBF for children predisposed to allergy or asthma Describe why the duration of EBF should be 6 months Describe why solid foods should be avoided in the first 6 months	Express confidence in giving 6 m-EBF (technically)	Expect that 6 m-EBF will decrease child’s risk for asthma-allergies Describe the importance of preparation for exclusive 6 m-EBF	

Talk about 6 m-EBF intention with care providers (e.g., midwives)	Describe the relevance of 6 m-EBF for children predisposed to allergy or asthma			

Decide before delivery to 6 m-EBF after delivery	Describe health–social advantages of 6 m-EBF Describe benefits of EBF for mother and child Describe anticipated difficult (physical, social, and work) situations related to 6 m-EBF	Express confidence in dealing with people not in favor of 6 m-EBF	Expect that giving 6 m-EBF is hard but achievable Express favorable attitude toward the importance of 6 m-EBF for mother and child Expect that environment can be convinced about the need to postpone solid food	Expect that partner will agree and support 6 m-EBF Anticipate social criticism about 6 m-EBF Minimize unfavorable opinions of others

Document questions to be asked during the prenatal home visit by project staff	Describe questions to be asked during the prenatal home visit by project staff			

Start and continue 6 m-EBF after delivery (on demand)	Describe that EBF needs to be learned Describe that an EBF-child has its own feeding schedule Describe the signals that child wants to be fed Describe what to plan for to EBF at work Describe what to do with physical complaints of mother or child Describe sources of help	Express confidence in the ability to breastfeed	Be convinced that EBF will be enough for a child to grow on	Expect positive remarks/support about 6 m-EBF by partner Minimize unfavorable opinion of others

Counteract social criticism toward 6 m-EBF		Express confidence in the ability to recognize and counteract social criticism		Expect that recognizing social criticism as risk situation helps to minimize its impact on mother Expect that other women who BF and postpone solid food go through the same experience

Buy or hire and use a breast pump to express breast milk	Describe where to buy or hire a breast pump Describe how to use a pump to express breast milk	State confidence that they can express milk with a breast pump	State positive feelings toward expressing breast milk	

Transport, store, and prepare breast milk safely	Describe how to safely transport, store, and prepare expressed breast milk	Express confidence to adequately transport, store, and prepare expressed milk	

Monitor child development (length and weight)	Describe how to use the growth curve for EFB child Describe that the growth curve of an EBF child deviates from a bottle fed child	Express confidence in assessing child’s progress in length and weight		Rely on the growth curve for EBF children when at infant well center that uses general population grow curves

Remind environment to not give solid food to a child		Express confidence that she can refrain others to give child solid food		Describe expectation that partner will support refraining others to give child solid food

Give solid food after 6 m-EBF	Describe how to introduce solid foods after 6 m-EBF			

**Table 2:** Partial matrix of change objective for partners of women that provide exclusively breastfeeding for 6 months (6 m-EBF).

Performance objectives before delivery	Knowledge	Skills and self-efficacy	Outcome expectations	Attitude
Partner will				
Express appreciation to mother who intends to 6 m-EBF Acquire information to prepare for 6 m-EBF (including the postponement of solid food)	Describe health and social advantages of 6 m-EBF (and postponement of solid food) Describe anticipated difficult (physical and social) situations related to 6 m-EBF and postponement of solid food Recognize the importance of partner for successful 6 m-EBF and postponement of solid food	Express confidence in supporting mother that gives 6 m-EBF (technically) Express confidence in dealing with people not in favor of 6 m-EBF and/or postponement of solid food	Expect that 6 m-EBF will decrease child’s risk for asthma-allergies	Express favorable attitude toward the importance of breastfeeding for mother and child Express favorable attitude toward the importance of postponement of solid food

**Performance objectives after delivery**				

Stand by mother who receives criticism because of 6 m-EBF Help to find solution for (physical) problems		Express confidence in dealing with people not in favor of 6 m-EBF Express confidence in supporting mother that gives 6 m-EBF (technically)		

### Steps 3 and 4: Program Design and Program Production

The eight women who pretested the brochure were multiparous women. Two of them had no experience with breastfeeding, three women breastfed an earlier child for 2–3 months, and three women breastfed an earlier child for more than 6 months. The results of the plus–minus method showed that the overall opinion about the brochure was very positive. The women thought that the brochure was interesting to read, attractive, and contained not too much or too little information. The used language appeared to be clear, and women appreciated examples and pictures. Despite the overall positive comments, the women had several suggestions to improve the brochure. Some women would like to have more information in the brochure about expressing milk and more focus on the social pressure of the environment. Other suggestions were a different title of the brochure and more pictures. We used information from these pretests to revise the program materials prior to implementation.

We present Steps 3 and 4 together so that the reader can readily understand the relations among theoretical behavior change methods, how the methods were delivered to parents and what specific topics and communication messages were conveyed. Table 3 presents these elements and shows how the theoretical methods match the types of change objectives they were intended to influence.

**Table 3:** Examples of objectives and methods for changing determinants.

**Determinant knowledge and unsupportive social norms**			

**Change objective**	**Methods**	**Parameters**	**Example**

*Performance objective* Recognize and counteract social criticism toward 6 m-EBF *Knowledge change objective* Describe anticipated difficult (physical and social) situations related to 6 m-EBF	Information about others (dis-) approval Resistance to social pressure Scenario-based risk information: scenarios can provide information on experiential stories about a potential future risk situation and/or how people have come to a solution (success frame) Modeling: providing a fitting model showing how beneficial behavior may be formed and is reinforced for the desired action.	Positive and negative expectations are available in the environment Commitment to earlier intention, psychological inoculation against pressure Credible scenario with a cause and an effect; can be fictional or experiential stories. Most effective when individuals produce their own scenario or when several scenarios are offered Recognition, recall, self-efficacy and skills, support of model; bonding, relatedness with the model, coping rather than mastery model	Application: experiential narrative in booklet Female model saying: “At one point I got to handle all kinds of comments from others. “*You cannot feed a child for 6 months only with breast milk*!” I found it very annoying that I constantly had to defend myself against all those people who think they know better. Even my family doctor started to tell me that I actually should start with adding fruit snacks at 4 months. Luckily, I was not be put out by it. I kept explaining every time why I choose for 6 months exclusively breastfeeding for my child. Most people still think it is abnormal what I do but, eventually, they stopped this discussion. It amazed me that I was considered being out of the ordinary because I opted for 6 months exclusively breastfeeding. I did not expect this.”

**Determinants knowledge and attitude**			

**Objective**	**Methods**	**Parameters**	**Application**

*Performance objective* Provide breast milk on demand *Knowledge change objective* Know that an EBF-child has its own feeding schedule *Attitude change objective* Be convinced that EBF will be enough for a child to grow on	Self-monitoring Encourage individuals to keep a record of particular behaviors Self-reevaluation	The monitoring must be of target behaviors related to objectives. Preferable an objective standard should be used to assess target behaviors. The collected data must be processed, evaluated, aid decision-making, action selection, and execution. The reward must be reinforcing to the individual. Feedback	Mother is instructed in the text to monitor when she breastfeeds and how often per 24 h, to monitor weekly weight gain; to check volume and number of diapers and several other indicators to evaluate whether the child is drinking (e.g., growing) enough

To change the determinants in step 2 and enable the performance objectives, we used a number of theoretical change methods from different theories. For example, looking at the first row of Table 3, we used “Modeling,” which is derived from Bandura’s social cognitive theory (21). The key messages the model expressed, tied to this theoretical method, were: (1) you might encounter negative reactions in relation to postpone solid food for 6 months; (2) you can explain why you do it; (3) if you stick to your own opinion, the discussion will diminish; (4) people will not change their attitude most times; and (5) do not be surprised, be prepared.

The key messages of the program were related to the central theme that exclusive breastfeeding (including the postponement of solid food) is the optimal and most normal/natural nutrition for newborns, but that it is hard work, and several barriers can make you quit before the intended 6 months. Therefore, preparation is needed. Presenting messages like this should increase the likelihood that readers process the content actively and stimulate an increase in their knowledge about the subject (22). Since knowledge alone is no guarantee for behavior change, it is important to provide the reader with the required skills to perform the behavior (21). Therefore, the booklet contained skill-based practical pictures (pictures with step by step guidance on how to latch a baby to the breast) alternated with the information on breastfeeding. Furthermore, we chose to combine written information with interpersonal communication. In our efficacy trial, the research staff created an opportunity for a dialog about breastfeeding and postponement of solid foods during the home visits (Table 4). For the broader implementation, the program is intended to be used in primary care, for instance, by midwives who see pregnant women frequently before and also sometime after birth. The home visitor and parents-to-be went through the written information together during the first home visit. Between the first and second visit, the women and their partner had the opportunity to read the brochure themselves and ask questions during the second visit. Moreover, the goal to create more equal partners in the conversation about breastfeeding (empowerment) could be addressed by this strategy. The interpersonal contact between the home visitor and parents was also aimed to review the information that had been read by the couples and to check understanding, clarify, repeat, and reinforce key messages (23).

**Table 4:** Content of breastfeeding booklet discussed during home visits and content of the home visit manual.

Main topics per period discussed in the booklet		Main topics addressed during home visits’ in manual
Introduction	Introducing four target group representatives (three women and one man) who share their personal experiences in the booklet. Central theme: breastfeeding is a challenge; social criticism is common, so be prepared!	Checklist of issues to be addressed during the phone call to make an appointment Protocol first home visit (3–4 months of pregnancy): use the checklist of materials needed, use the checklist of topics to address, such as information on the study, on asthma prevention (hand over two asthma booklets), preventive measures to be taken to reduce house dust mite, smoke exposure, and for those without breastfeeding intention, information on hypo-allergic formula. Focus on pros and cons of breastfeeding and postponement of solid food, and breastfeeding intention Protocol second home visit (seventh month of pregnancy): use the checklist of materials needed, discuss preventive measures taken, repeat information on pros and cons of breastfeeding and postponement of solid food, and breastfeeding intention. Bring social criticism to the attention Protocol third home visit (2–4 weeks postpartum): use the checklist of materials needed, discuss preventive measures taken, inquire after breastfeeding behavior, repeat information on pros and cons of breastfeeding and postponement of solid food, and breastfeeding continuation. Inquire after experience with social criticism, preparation for work and experience with expressing milk/breast pumps
During pregnancy	Why “breast is best” for infant and mother Specific health benefits for families predisposed to asthma or allergy Breastfeeding duration Breastfeeding and the use of asthma medication The special role of the father as a coach for the mother What to do when no breastfeeding support is available in hospital Breastfeeding and Cesarean section	
The first few weeks after delivery	How breastfeeding works: good breastfeeding positioning, infant latching, frequency of feeding Common breastfeeding myths How to check if the child receives enough breast milk. How to overcome sore and inverted nipples, breast engorgement and mastitis Cow milk allergy and diet of the mother What to expect from health professionals regarding advice on breastfeeding Breastfeeding in public Breastfeeding and anticonception Coping with response from others	
A few months after delivery	When to try the bottle with breast milk? What if your child refuses the bottle? Expressing milk, how does it work? How to restore expressed milk Breastfeeding/expressing milk during work time and the law Alternatives to breastfeeding Introducing solids after 6 months Coping with response from others	
Additional	Phone numbers of lactation, organizations/consultants, useful websites, and further reading options	

Furthermore, risk awareness messages (your child might be predisposed to asthma) underlined the child’s vulnerability to the threat (22), but at the same time, we presented an effective solution that women were able to perform (increase self-efficacy) breastfeeding exclusively for 6 months. This combination of awareness messages aimed at increasing threat perception and efficacy information is more likely to stimulate people to uptake the desired health behavior (24, 25). In our attitude strategy, we explicitly paid attention to existing beliefs for women with previous brief breastfeeding experience, since these women are at risk of not continuing for longer periods. For this group, we acknowledged that it was difficult to breastfeed, but being better prepared this time, they could do it. We included persuasive messages to accomplish positive breastfeeding attitudinal beliefs in first-time mothers. The models in the booklet were chosen for several reasons, but mainly to ensure that women identified with them. The four role models (selected by women of the target group for identification) illustrated the information and provided real-life experiences related to adherence to breastfeeding. These experiences were inspired by focus group stories. The goal was for the models to stimulate positive attitudinal beliefs, increase feelings of self-efficacy, but also to provide warning stories to prepare families for the social pressure they can expect and examples of coping responses (21). Another important step was to pretest the program in members of the target population before actual implementation of the intervention. The eight women who pretested the booklet were multiparous. Two of them had no experience with breastfeeding, three women breastfed an earlier child for 2–3 months, and three women breastfed an earlier child for more than 6 months. The results of the plus–minus method showed that the overall opinion about the booklet was very positive. The women commented that the brochure was interesting to read, useful and contained not too much or too little information. The language in the brochure appeared to be clear, and examples and pictures were appreciated. Despite the overall positive evaluations, the women had several suggestions to improve the brochure. For example, one woman liked to have more information about expressing milk and more examples on how to handle the social pressure of the environment in the brochure. Other suggestions were a different title of the brochure and more pictures.

### Step 6: Planning for Evaluation

A description of the study design has been published elsewhere (26). At 6 months, the percentage of women breastfeeding exclusively was significantly higher in the intervention group than among the control group, respectively, 48 versus 27%; odds ratio 2.91; 95% confidence interval (1.10–7.71) (*p* < 0.03) (26). Substantial increases in knowledge and more positive beliefs regarding ongoing breastfeeding were revealed, especially directly after exposure to the program, in the intervention group compared with the controls. Perceived self-efficacy and women’s positive emotions toward ongoing breastfeeding increased and perceived support for breastfeeding decreased in both groups. The intervention group reported a higher level of perceived social pressure to bottle feed and reported to know more other women that breastfeed than the control group (9).

Parents in the intervention group completed a questionnaire about the usefulness of the breastfeeding booklet and home visit. Almost all women (98%) read the booklet in whole or parts of it. The booklet was evaluated accessible, easy to read, and attractive. The overall appreciation of the program on a 10-point scale was rewarded with an 8.1 (range 6–10). The main comments reflected a generally positive view of the program.

## Discussion

In this paper, we described the development, theoretical behavior change methods, and content of a program that effectively increased the proportion of women who were able to sustain breastfeeding (26). The program was delivered through a combination of pre- and postnatal home visits and a corresponding theory-based booklet. The program increased the long-term duration of exclusive breastfeeding; the experimental group significantly outweighed the control group in days that their child received exclusive breastfeeding and more children in the experimental group received exclusive breastfeeding for 6 months (26). An explanation for the success of our breastfeeding program could be found in the significant impact on determinants of breastfeeding that were targeted by our program (27–30). Besides, during a meeting with parents in which they reflected on the program they had received, parents stated that the prenatal preparation for the unexpected negative attitudes and criticism from others had been helpful to continue breastfeeding. A recent study stressed the negative influence of social criticism on breastfeeding behavior as well (31). In the Netherlands, only one other study evaluated the effectiveness of a breastfeeding promotion program. This program aimed to increase the breastfeeding continuation until at least 3 months with a health counseling training for the caregivers in postpartum care, in order to enhance the cooperation between caregivers and continuity of care, and early signaling of breastfeeding problems and free lactation consultancy (OR 0.82, 95% CI 0.58–1.14) (32). Emphasis on educating the postpartum health professionals could be a wrong assumption since women chose early in pregnancy if they will breastfeed their child and can already benefit from prenatal support and education. Putting effort in preparing and educating the women starting from mid-pregnancy seems more logical than to train health professionals, which the women will see later in their pregnancy or in some cases even postpartum.

Many breastfeeding promotion programs to extend the duration of breastfeeding have been evaluated, but the majority of the studies are not comparable with each other due to several different basic assumptions (32–34). For example, most studies focus just on education or on support only or provide only written materials. Additionally, the timing points of contact moments fluctuate (pre- or postnatal or both), contact formats vary (the usage of group sessions or individual contacts, face-to-face, or just telephone contacts), and frequency of contacts differ (32). Two studies (35, 36), for instance, showed that only offering women written material was not successful, the difference between the success rate of the intervention and control group was only 7 percent after 6 months in one study (59 versus 52%), and 4% in the other study (48 versus 44%). Sikorski et al. (33) concluded in their systematic review that offering extra support leads to a higher proportion of women who breastfeed exclusively the first 6 months. The studies that were compared used different supportive strategies and trained volunteers or professionals to offer the breastfeeding support. A meta-analysis of four trials, which made use of the WHO/UNICEF training (a training to educate health professionals) showed significant benefit in prolonging exclusive breastfeeding (RR 0.70, 95% CI 0.53–0.93), but results were considered highly heterogeneous. Analysis of studies reporting a predominantly face-to-face intervention showed a statistically significant benefit (RR 0.86, 95% CI 0.78–0.94; eight trials, 2,044 women), whereas those using mainly telephone contact had almost a similar RR that was not statistically significant (RR 0.92, 95% CI 0.78–1.08, five trials, 1,168 women). Guise et al. (34) performed a systematic review and included studies, which originated in the primary care setting and contained a concurrent control group. They concluded that the effect regarding long-term breastfeeding practices (4–6 months) was the largest when breastfeeding education was combined with support. Hence, a recent Cochrane review on antenatal breastfeeding education to increase breastfeeding duration concluded that the evidence supporting any prenatal breastfeeding education to improve (1) the initiation of breastfeeding, (2) the proportion of women giving any breastfeeding or exclusively breastfeeding at 3 or 6 months, and (3) the duration of breastfeeding is still inconclusive (37). In addition, methodology problems in studies to evaluate breastfeeding interventions include short follow-up periods (less than 6 months), poor quality trials due to substantial baseline differences or lack of adjustment to confounders, and the vague definition of the outcome measure (7). In most reviews, the impact of whether educational interventions were based on behavioral change theory-based is largely ignored.

In conclusion, strong points of our program are the systematic development based on theoretical models, pre- and postnatal home visits, intervention starting in mid-pregnancy, and the supportive role of the home visitor, stressing partner support and resistance to social influence in combination with the written material.

### The Use of IM

Intervention Mapping helps programs development and it also helps to describe existing interventions explaining what is “inside the black box” (12). In this project, IM guided a thorough analysis of the problem, program goals, and performance objectives, and determinants, which led to the development of an education program to promote exclusive breastfeeding by carefully describing the various steps and tasks of the IM protocol. IM is a complex and time-consuming process, reflecting the difficulty of changing (health) behaviors. For instance, during program development, describing performance objectives forced us to describe the sequence of actions that are needed to safely transport expressed breast milk from work to home, which led to a more detailed informational section (e.g., storage temperature, cleaning containers) than we would have written otherwise. Also, having to think about the core messages of our program helped us to better formulate the two major themes of the program “Exclusive breastfeeding is not easy at all, but if you prepare yourself during pregnancy, you can do it,” and “Fathers’ support is essential to succeed in exclusive breastfeeding.” IM also forces developers to carefully consider methods and parameters of use. For instance, in our study, we let the target population select the pictures of the models the target group could identify with (parameter for use) instead of selecting them ourselves. Furthermore, our participatory approach to program development enabled us to identify the importance of the support the partners during our needs assessment, which helped us to consider mothers and partners as a breastfeeding team, instead of considering partners as an external influential person that might support mothers providing breastfeeding.

Above examples illustrate that IM can be complex, elaborate, tiresome, expensive, and time-consuming. However, IM also assists in bringing the development of interventions to a higher level, and it helps intervention planners develop the best possible intervention.

## Implications for Practice

At a national level, important organizations such as the professional organization for midwives and the health home care services adopted the national feeding guidelines for newborns to promote exclusive breastfeeding for the first 6 months. Also, the Dutch government promotes breastfeeding for 6 months because of the many proven health benefits for mother and child. Our effective breastfeeding promotion program was evaluated positively by participating families who graded the program with an eight on a scale from 0 to 10. We believe this program holds a promise for future care to coach women and their partner, pregnant of a predisposed child, toward adherence to breastfeeding for 6 months and improve health in high-risk newborns. Therefore, an attempt must be made to implement the program in the daily practice of health professionals, especially in prenatal care.

## Ethics Statement

Medical ethics review committee Maastricht University Medical Center and Maastricht University.

## Author Contributions

IM and BG are the investigators of this study and developed the breastfeeding intervention. KB was the external consultant on Intervention Mapping. All authors contributed to writing the article submitted. KB passed away in February 2016.

## Conflict of Interest Statement

The authors declare that the research was conducted in the absence of any commercial or financial relationships that could be construed as a potential conflict of interest.

## Footnotes

^1^Exclusive breastfeeding is defined according the WHO definition (5). Nutritional advice is subjected to changes in scientific research outcomes. Currently, the postponement of solid foods is advised until 4–6 months after birth (6).
